# Optimizing Treatment Strategies for Distal Radius Fractures in Osteoporosis: A Comparative Review

**DOI:** 10.3390/medicina60111848

**Published:** 2024-11-10

**Authors:** Eric J. Gullborg, Jason H. Kim, Caitlin M. Ward, Xavier C. Simcock

**Affiliations:** Department of Orthopaedic Surgery, Rush University Medical Center, Chicago, IL 60612, USA; jason_h_kim@rush.edu (J.H.K.); caitlin_m_ward@rush.edu (C.M.W.); xavier.simcock@rushortho.com (X.C.S.)

**Keywords:** distal radius fractures, osteoporosis, operative management, nonoperative management, age

## Abstract

Osteoporosis is a common yet underdiagnosed condition that increases the risk of fractures, contributing to substantial morbidity, mortality, and healthcare costs. Distal radius fractures (DRFs) are some of the most common fractures associated with osteoporosis and often precede more severe fractures. Managing DRFs in patients with osteoporosis can be a challenge due to altered bone quality, which can affect healing and surgical fixation. This review examines both operative and nonoperative management strategies for DRFs in osteoporotic patients, emphasizing the importance of individualized treatment. Surgical interventions, like open reduction and internal fixation (ORIF) with plating, can facilitate early mobilization and improved alignment, especially in more active patients. However, osteoporosis poses risks such as hardware failure, infection, and malunion, calling for careful patient selection. Conversely, nonoperative management may be more suitable for patients with lower functional demands or higher surgical risks, despite the increased risk of malunion. By adapting treatment strategies to individual patient characteristics, orthopedic surgeons can optimize outcomes, minimize complications, and potentially prevent future fractures. Both operative and nonoperative treatments can yield positive outcomes when personalized to the patient’s needs.

## 1. Introduction

Osteoporosis is the most common bone disease worldwide with an overall prevalence estimated to be around 18.3% to 19.7% [1]. This condition is characterized by decreased bone density, bone pain, and increased risk of fractures, imposing significant morbidity and mortality for patients, and presents a financial burden on the healthcare system. Osteoporosis and its sequelae disproportionately effect older patients, as one in three women and one in five men over the ages of 50 years will experience a fracture attributed to osteoporosis [2]. The prevalence of osteoporosis in the elderly has notably increased from 9.4% in 2007–2008 to 12.6% in 2017–2018 and continues to increase as the elderly population grows [3].

Distal radius fractures (DRFs) are second in prevalence only to hip fractures and are the most common fragility fracture experienced by women over 50 years of age [4,5,6,7]. DRFs typically occur earlier in life compared to other fragility fractures and are the most common fractures of the upper extremity [4,5,6,7]. The highest incidence of DRFs is seen in patients aged 60–69 and the biggest predisposing risk factor is osteoporosis [8]. The association between osteoporosis and DRFs has been well established throughout the literature; for instance, it has been found that 68% of women over the age of 60 with DRFs have osteoporosis [6].

DRFs are important in the context of osteoporosis because they often precede more serious fractures, such as hip fractures. Patients who have suffered a DRF have been shown to be at a five-times-higher risk of sustaining a hip fracture within one year compared to their counterparts [9]. Given their high prevalence, DRFs can serve as a crucial focus for understanding the broader impact of osteoporosis on fracture treatment and outcomes. Osteoporosis patients present unique challenges in fracture management due to the altered biology of their bones. Decreased bone mass, reduced cortical thickness, and more porosity can complicate both fracture healing and the surgical fixation process, leading to higher rates of complications [10]. The goal of this review is to evaluate and compare the current literature regarding operative versus nonoperative treatment of DRFs in osteoporotic patients, and consolidate our findings into recommendations for optimizing outcomes in this patient population. 

## 2. Operative Management of DRFs in Patients with Osteoporosis

Since the early 2000s, surgical intervention, particularly open reduction and internal fixation (ORIF) with plating, has become a common choice for managing DRFs in osteoporotic patients [8,11]. Effective operative management of DRFs in osteoporotic patients depends on the selection of appropriate fixation techniques. Although the use of volar locking plates has been the most popular in past years, dorsal bridge plates and hemiarthroplasty can be performed and may offer distinct advantages [12]. Due to the altered bone quality in osteoporotic patients, careful consideration must be given to the risks and benefits associated with each method of surgery.

### 2.1. Benefits of Operative Management 

Several studies support the use of ORIF for DRFs in patients with osteoporosis. It has been established that volar locking plates are generally very effective even in osteoporotic bone, as they can provide sufficient stability needed for fracture realignment [13]. Choi et al. found that using volar locking plates for ORIFs in postmenopausal women with osteoporosis yielded similar functional outcomes to nonosteoporotic patients, suggesting that this surgical technique may mitigate some of the negative impacts of osteoporosis postoperatively [14]. Moreover, Lee et al. retrospectively reviewed women older than 50 years old with DRFs treated with volar plates, comparing outcomes between those with and without osteoporosis. They found no differences in clinical outcomes between the two groups, reinforcing that volar plate fixation is effective even in osteoporotic bone [15]. Even so, internal radiocarpal distraction plating (bridge plating) has been found to be a suitable alternative for osteoporotic patients with complex, high-risk DRFs [16]. This surgical option can anchor the plate in stronger bone, bypassing the fracture site which may not reliably hold screws due to the presence of osteoporosis [16]. Recent reviews systematically looked at dorsal bridge plating, further highlighting their benefits of providing stable fixation in severe DRFs, especially in patients with poor bone quality [17,18]. It was found that this technique allowed early weight-bearing, early mobilization, and achieved satisfactory functional outcomes such as range of motion, grip strength, Quick Dash score, and overall patient satisfaction [17,18]. This was further reinforced by Defazio et al.’s recent retrospective study, which analyzed 43 dorsal plate fixations and found few complications and good functional outcomes overall [19].

Wrist hemiarthroplasty has also emerged as a viable surgical option for patients with “irreparable DRF in the elderly” [20]. Fractures among the elderly with poor bone quality may not be effectively treated with plating due to severe impaction and comminution [20]. A study followed 17 wrists with an average age of 79 post wrist hemiarthroplasty and found satisfactory functional and radiographic results in all patients, with a final follow-up showing an average VAS pain score of 1/10 and functional range of motion [20]. A case report of a 73-year-old patient with a complex intraarticular DRF and severe osteoporosis further showcases the benefits of a wrist hemiarthroplasty. The patient achieved significant pain relief and stability observed over 6.5 years post-surgery [21]. This is all important to highlight as this procedure does not rely on screw purchase in weakened bone, thus avoiding complications that osteoporosis may bring in procedures such as volar plating. Wrist hemiarthroplasty is a surgical technique that allows for sustained functionality and satisfaction even in cases of severe bone fragility.

Osteoporotic patients are at significant risk for loss of reduction and malunion due to their inherent instability [8]. However, trends towards increased use of internal fixation in the elderly reflect a preference for achieving better anatomical alignment and facilitating early mobilization [22]. Internal fixation can provide the necessary stability for patients to return to activities of daily living sooner, potentially minimizing the prolonged immobilization that could exacerbate further osteopenia [23,24]. A prospective randomized trial that compared ORIF to closed reduction and percutaneous pin fixation for treatment of DRFs demonstrated that patients treated with ORIF had significantly better outcomes in the first 12 weeks postoperatively, although outcomes became similar by one year [25]. This shows that osteoporotic patients can benefit from early mobilization through operative treatment and decrease the risk of further osteopenia, joint stiffness, and functional decline.

Osteoporosis affects approximately 6.3% of men and 21.2% of women over the age of 50 globally, suggesting that approximately 500 million men and women worldwide may be affected [26]. Given this statistic, it is very likely that many patients undergoing DRF surgery are impacted by osteoporosis even if not explicitly screened in various studies. For instance, when looking at adults older than 60 with a DRF, increased chronological age has been associated with better functional outcomes in surgically treated patients at all timepoints assessed through a one-year period [27]. Additionally, patients with higher activity levels also demonstrated improved functional outcomes following surgery, indicating that physiological factors can play a vital role in recovery [27]. Another study found no significant differences in functional outcomes between patients aged 65–74 and patients over the age of 75 who underwent ORIF for DRF, emphasizing that age should not be the sole determinant in choosing surgical options [28]. Patients over 75 who are found to be physiologically younger and independent can achieve functional outcomes similar to younger cohorts when treated surgically [28]. 

### 2.2. Risks of Operative Management 

Despite the benefits of operative treatment, osteoporosis presents significant biological challenges that may affect surgical outcomes. Osteoporotic bones are characterized by reduced bone mass, decreased cortical thickness, and increased porosity, which can impair effective fracture repair [10]. These structural changes compromise the bone’s ability to support hardware, causing poor screw purchase and increasing the risk of hardware failure, malunion, fracture displacement, and instability [29,30,31]. Internal fixation in osteoporotic bone overall has been found to have failure rates ranging from 10% to 25% due to decreased holding power of plate and screw constructs [29]. Bone failure, rather than actual implant breakage, is cited as the primary reason for failure [29]. Despite advancements in fixation devices like volar locking plates, the risk of screw loosening or poor fixation still exists due to the overall fragile bone structure [13]. Furthermore, osteoporotic bones are more susceptible to surgical site infections, as low BMD has been identified as an independent risk factor for such infections following orthopedic procedures like lumbar fusion surgery [32].

FitzPatrick et al. found that after 12 months, osteoporotic patients undergoing ORIF for DRFs had significantly worse functional outcomes per the Disabilities of the Arm, Shoulder, and Hand (DASH) scores and higher complication rates, including instability and malunion, when compared to nonosteoporotic patients [33]. This argues that while surgical intervention can offer better early outcomes, the risk of complications in the long term remains high. A downfall of FitzPatrick et al.’s study is its relatively small effect size, and Tuaño et al. attempted to address this by using a large-scale commercially available healthcare database [31]. Their results also revealed that both osteopenia and osteoporosis were associated with increased risk of hardware failure, surgical site infection, and malunion one year after surgery. This suggests that osteoporosis can significantly hamper postoperative recovery due to the fragility, instability, and poor healing capacity of osteoporotic bones [8]. It must be stated that it remains controversial whether complications are solely due to osteoporosis because of its association with increased age and age-related disorders [10]. Moreover, it was found that patients 70 years and older treated with volar locking plate fixation had significantly higher levels of pain at final postoperative follow-up when compared to a similar group who were treated nonoperatively [34]. Although the study did not specifically screen for osteoporosis, the finding suggests that older adults, who are more likely to have osteoporotic bones, might not experience expected pain relief from surgical intervention. This emphasizes the importance of considering not just bone health, but also age-related factors and patient goals when deciding on operative management.

## 3. Nonoperative Management of DRFs in Patients with Osteoporosis

Nonoperative management, typically involving immobilization with casts or splints, has been a traditional treatment choice for DRFs and continues to remain a viable option. This is generally preferred for patients who have stable fractures, limited functional demands, or those with higher risks for surgical complications. However, as with surgical management, the success of nonoperative management in osteoporotic patients can be limited by the biological effects of osteoporosis on bone healing, leading to potential complications such as malunion, nonunion, or loss of reduction [35,36].

### 3.1. Benefits of Nonoperative Management 

Patients with lower functional demands and limited mobility with the primary goal of pain relief can benefit from conservative approaches [35]. Testa et al. found no significant differences in functional outcomes and pain when comparing patients over the age of 65 treated with ORIF to those treated conservatively with immobilization [37]. Conservative treatment may be an appropriate option for elderly osteoporotic patients when fracture stability is maintained. Boymans et al. evaluated patients one year following conservative treatment with cast immobilization, with or without closed reduction [38]. Functional outcomes assessed did not correlate with BMD, suggesting that BMD measurements may not be a reliable predictor of functional recovery in nonoperative cases [38]. This study illustrates that conservative management can still result in favorable outcomes in patients with and without osteoporosis.

Studies show that in elderly patients with low-energy DRFs, functional outcomes can be satisfactory even in the presence of visible deformity, primarily because these patients tend to have lower physical activity levels, lower functional demands, and reduced soft tissue involvement [8,36]. A study that compared operative treatment to casting in patients older than 70 years old with DRFs found that there were no significant differences between range of motion, grip strength, DASH, and patient-related wrist evaluation scores at final follow-up, despite having notably worse radiological outcomes [34]. A total of 77% of these patients had an obvious clinical deformity; however, none of these patients were found to be dissatisfied with their appearance [34]. This idea is reinforced by a recent comprehensive review that concluded that nonoperative management of DRFs in the elderly can yield outcomes comparable to surgery in terms of function and pain relief, even with a residual deformity present [39]. The review noted that deformity occurs in about 30% of nonoperative cases but has noninferior results when compared to surgical outcomes [39]. Furthermore, a recent cohort study followed 73 patients aged 65–88 over a 12-month period comparing outcomes between those treated nonoperatively versus those who underwent surgery of DRFs. The study found no differences in functional outcomes other than grip strength between surgical and nonoperative groups at both 6-month and 12-month follow-up [40]. Interestingly, this study found that 78% of nonoperative patients with a visible deformity present reported satisfaction with both functional and cosmetic outcomes at final follow-up [40]. While osteoporosis was not explicitly addressed in these studies, the findings suggest that many older patients, with or without osteoporosis, can achieve goals consistent with pain relief and preservation of function, all while avoiding the risk associated with surgery and maintaining acceptable quality of life.

Moreover, Azad et al. found that the overall complication rate for DRFs treated nonoperatively was 5.4% which is significantly lower than the complication rate seen in operative treatment of 9.4% [8]. They found that the group that received nonoperative treatment had significantly lower rates of every complication except for malunion when compared to the operative group [8]. A retrospective cohort study with 311 distal radius fractures specifically looked at delayed union and nonunion in patients with and without osteoporosis. The study found no cases of delayed or nonunion in the DRFs regardless of osteoporosis status [41]. Despite lower bone quality, healing outcomes in the osteoporotic group were comparable to those in patients with normal bone density, indicating that osteoporosis may not be as critical a factor in the healing of DRFs as initially expected. This is further proven in a study by Arora et al., which demonstrated that patients treated nonoperatively with casting had no complications at all, with the exception of CRPS versus a complication rate of 13% in the surgical group [34]. 

### 3.2. Risks of Nonoperative Treatment

Despite having its advantages, nonoperative treatment can still present significant challenges in osteoporotic patients. The fragility of osteoporotic bone can make it difficult to maintain fracture alignment through casting or immobilization alone, increasing the risk of malunion and loss of reduction [35,36]. The decreased bone mass and disorganized trabecular structure make it challenging for immobilization techniques to provide sufficient stability. This increases the likelihood of long-term complications such as joint stiffness, functional decline, reduced range of motion, and long-term disability, which can ultimately impact patient-reported outcomes [35]. It was found in a recent study, looking at adults over 50 years old with DRFs, that osteoporotic patients demonstrate significantly more displacement characteristics such as greater dorsal angulation and increased ulnar variance [42]. This increased fracture displacement could complicate nonoperative management by exacerbating instability, as maintaining reduction may be challenging in these cases. Furthermore, this study found that such structural deformities can be linked to triangular fibrocartilage complex (TFCC) injuries, which may further worsen joint instability if left unaddressed [42]. It is thought that the increased displacement in osteoporotic fractures can contribute to this ligamentous injury [42]. This highlights the need for careful patient selection and close monitoring during nonoperative management. Nonoperative management of DRFs need accurate radiographic assessment of fracture reduction. A recent study evaluated the reliability of radiographic measurements commonly taken to assess casting position in DRFs. The study showed high inter- and intraobserver agreement in certain areas like radial inclination, shortening, and dorsal/volar tilt; however, the study also found poor agreement for intra-articular gap and step measurements [43]. This is significant as this may question the reliability of measurement techniques in guiding the decision for nonoperative management in DRFs. This could place patients at risk for complications, as fractures needing surgical intervention could be mistakenly treated nonoperatively. This is especially important in osteoporotic bones where precise alignment is crucial in avoiding malunion.

As Azad et al. found, even though nonoperative treatment had lower overall complication rates compared to surgery, the increased risk of malunion was found to be higher [8]. Some elderly individuals with low functional demands may tolerate deformity; however, those with higher activity levels or those relying on their motor skills may experience significant deficits in strength, mobility, and range of motion [36]. Moreover, it has been established that bone structure naturally adapts to provide adequate strength for daily activities of living, and prolonged immobilization can result in reduced mass of bone over time [23]. Therefore, in the nonoperative treatment of a DRF, extended immobilization of already osteoporotic bone may further exacerbate the patient’s compromised bone health. In fact, a recent retrospective study demonstrated that surgical treatment had lower odds of developing disuse osteopenia within six weeks compared to nonoperative treatment for a DRF, although it is important to note that only increasing age was found to be a significantly associated factor [44].

## 4. Discussion

Although a well-established association exists between osteoporosis and DRFs, the optimal treatment options for such fractures in osteoporotic patients still remain circumstantial and dependent on the individual. The evidence provided suggests that both nonoperative and operative treatment modalities can provide beneficial outcomes depending on the individual patient’s baseline function and goals. Much of the previous literature has focused on age as the primary factor when choosing a treatment plan, and while increased age may predict poorer outcomes under some circumstances, it is not the sole factor that should guide treatment decisions. It is clear that an individualized approach, one that considers each patient’s lifestyle, physical activity level, comorbidities, overall health status, and personal goals, must be considered when treating an osteoporotic patient with a DRF.

For physically active patients or those with high functional demands, our interpretation of the literature suggests that operative management, particularly open reduction and internal fixation, may provide the best chance for returning to preinjury activity levels. Early mobilization facilitated by ORIF can lead to superior functional outcomes in the short term [14,24]. Early mobilization is especially important in these patients as prolonged immobilization can further exacerbate bone loss and lead to further complications. The benefits of quicker recovery and minimizing decline in bone quality outweigh the associated risk for surgery in this subset of patients. Patients with osteoporosis and a DRF who are otherwise healthy and active should be given the opportunity for surgical management regardless of their age.

The literature also presents compelling evidence to support nonoperative treatment, particularly for more frail or inactive patients with osteoporosis and a DRF. The risks of surgery, such as hardware failure, infection, and increased pain, may outweigh the benefits of surgical treatment, especially in patients whose primary goal is pain relief rather than functional restoration. Nonoperative treatment offers lower complication rates, making it a more attractive option for patients who are at higher risk for surgical complications. While we recommend nonoperative treatment as an approach in select patients, it is important to recognize that this treatment is not without its risks of malunion, worsening osteoporosis, and loss of fracture alignment. Regardless, clinical deformity in nonoperatively treated patients does not always correlate with dissatisfaction or reduced functional outcomes [34]. This emphasizes the importance of aligning treatment goals with patient lifestyle and overall health status. We recommend nonoperative treatment in patients whose primary goals are pain relief and basic functional lifestyle goals.

Nonetheless, studies have shown that early osteoporosis screening and timely treatment are linked to fewer future fractures, thus stressing the need to make osteoporosis management a key part of care for patients with a DRF [45]. It has been found that only 25% of patients who present with a DRF undergo an evaluation for osteoporosis [46,47]. Recent findings demonstrate the significant diagnostic and treatment gaps in osteoporosis care, with one study finding a 53% diagnostic gap and 84% treatment gap among DRF patients [48]. These patients were eligible for osteoporosis treatment before their fracture and many of them had suboptimal vitamin D levels [48]. Orthopedic surgeons are faced with a unique opportunity to influence this health directive, as studies have shown that the rate of DEXA scans ordered, completed, and treatment option education provided by primary care physician increases to over 70% when initiated by an orthopedic surgeon [46]. Regardless of the chosen treatment approach, osteoporosis management should be emphasized at the forefront.

Despite the insights offered in this review, there are several gaps that still need attention in future research on this topic. Future studies should use standardized patient groups based not only on age, but also on several lifestyle factors such as BMI, activity level, comorbidities, and the severity of osteoporosis to assess the outcomes of various treatment modalities. Another key area of potential research is investigating how osteoporosis medications impact different fracture treatments, both nonoperative and operative, when looking at long-term outcomes. Understanding how these medications influence the rate of bone healing, risk of complications, and functional outcomes could significantly affect clinical practice and decision making.

## 5. Conclusions

In summary, osteoporosis plays a detrimental role in the bone-healing process following a distal radius fracture and can complicate treatment decision making. Clinically, the management of DRFs in osteoporotic patients requires that clinicians look beyond chronological age and initiate a tailored approach that considers a patient’s whole picture. Both operative and nonoperative options have been shown to result in beneficial outcomes. For more physically active patients, operative management has been shown to be most suitable, and for patients with limited baseline activity or those at higher risk for surgical complications, conservative management will be the better option. Nonetheless, early osteoporosis screening and timely treatment are linked to fewer future fractures, thus emphasizing the need to make osteoporosis management a key part of care for patients when dealing with a DRF.

## Data Availability

No new data were created or analyzed in this study. Data sharing is not applicable to this article.

## References

[1] Salari N., Darvishi N., Bartina Y., Larti M., Kiaei A., Hemmati M., Shohaimi S., Mohammadi M. (2021). Global prevalence of osteoporosis among the world older adults: A comprehensive systematic review and meta-analysis. J. Orthop. Surg. Res..

[2] Melton L.J., Atkinson E.J., O’Connor M.K., O’Fallon W.M., Riggs B.L. (1998). Bone density and fracture risk in men. J. Bone Miner. Res..

[3] Sarafrazi N., Wambogo E.A., Shepherd J.A. (2021). Osteoporosis or Low Bone Mass in Older Adults: United States 2017–2018. NCHS Data Brief.

[4] Lane N.E. (2006). Epidemiology, etiology, and diagnosis of osteoporosis. Am. J. Obstet. Gynecol..

[5] Karl J.W., Olson P.R., Rosenwasser M.P. (2015). The Epidemiology of Upper Extremity Fractures in the United States, 2009. J. Orthop. Trauma.

[6] Bahari S., Morris S., Lenehan B., McElwain J.P. (2007). “Osteoporosis and orthopods”: Incidences of osteoporosis in distal radius fracture from low energy trauma. Injury.

[7] Beringer T.R.O., Finch M., Taggart H., Whitehead E., Keegan D.A.J., Kelly J., Lee G., McKane R., McNally C., McQuilken M. (2004). A study of bone mineral density in women with forearm fracture in Northern Ireland. Osteoporos. Int..

[8] Azad A., Kang H.P., Alluri R.K., Vakhshori V., Kay H.F., Ghiassi A. (2019). Epidemiological and Treatment Trends of Distal Radius Fractures across Multiple Age Groups. J. Wrist Surg..

[9] Shoji M.M., Ingall E.M., Rozental T.D. (2021). Upper Extremity Fragility Fractures. J. Hand Surg. Am..

[10] Chandran M., Akesson K.E., Javaid M.K., Harvey N., Blank R.D., Brandi M.L., Chevalley T., Cinelli P., Cooper C., Lems W. (2024). Impact of osteoporosis and osteoporosis medications on fracture healing: A narrative review. Osteoporos. Int..

[11] Shauver M.J., Yin H., Banerjee M., Chung K.C. (2011). Current and future national costs to Medicare for the treatment of distal radius fracture in the elderly. J. Hand Surg. Am..

[12] Schindelar L.E., Ilyas A.M. (2021). Plate fixation of distal radius fractures: What type of plate to use and when?. Hand Clin..

[13] Konstantinidis L., Helwig P., Hirschmüller A., Langenmair E., Südkamp N.P., Augat P. (2016). When is the stability of a fracture fixation limited by osteoporotic bone?. Injury.

[14] Choi W.S., Lee H.J., Kim D.Y., Lee C.H., Lee B.G., Kim J.H., Lee K.H. (2015). Does osteoporosis have a negative effect on the functional outcome of an osteoporotic distal radial fracture treated with a volar locking plate?. Bone Jt. J..

[15] Lee J.I., Park K.C., Joo I.H., Jeong H.W., Park J.W. (2018). The effect of osteoporosis on the outcomes after volar locking plate fixation in female patients older than 50 years with unstable distal radius fractures. J. Hand Surg. Am..

[16] Vakhshori V., Alluri R.K., Stevanovic M., Ghiassi A. (2020). Review of internal radiocarpal distraction plating for distal radius fracture fixation. Hand.

[17] Labrum J.T., Ilyas A.M. (2021). Bridge plate fixation of distal radius fractures: Indications, techniques, and outcomes. Orthopedics.

[18] Fares A.B., Childs B.R., Polmear M.M., Clark D.M., Nesti L.J., Dunn J.C. (2021). Dorsal bridge plate for distal radius fractures: A systematic review. J. Hand Surg. Am..

[19] DeFazio M.W., Godfrey N., Offord E., Budis E., Olson N., Jones M. (2024). Distal radius fracture outcomes after dorsal spanning plate fixation. HAND.

[20] Herzberg G., Burnier M., Ly L. (2023). Role for wrist hemiarthroplasty in acute irreparable distal radius fracture in the elderly. Hand Clin..

[21] Holzbauer M., Bodell L.S., Froschauer S.M. (2022). Wrist hemiarthroplasty for complex intraarticular distal radius fracture in a patient with manifest osteoporosis. Life.

[22] Mauck B.M., Swigler C.W. (2018). Evidence-Based Review of Distal Radius Fractures. Orthop. Clin. N. Am..

[23] Cardozo C., Bauman W.A., Marcus R., Feldman D., Dempster D.W., Luckey M., Cauley J.A. (2021). Immobilization osteoporosis. Marcus and Feldman’s Osteoporosis.

[24] Orbay J.L., Fernandez D.L. (2004). Volar fixed-angle plate fixation for unstable distal radius fractures in the elderly patient. J. Hand Surg. Am..

[25] Rozental T.D., Blazar P.E., Franko O.I., Chacko A.T., Earp B.E., Day C.S. (2009). Functional outcomes for unstable distal radial fractures treated with open reduction and internal fixation or closed reduction and percutaneous fixation: A prospective randomized trial. J. Bone Jt. Surg. Am..

[26] Kanis J.A., McCloskey E.V., Johansson H., Oden A., Melton L.J., Khaltaev N. (2008). A reference standard for the description of osteoporosis. Bone.

[27] Jayaram M., Wu H., Yoon A.P., Kane R.L., Wang L., Chung K.C. (2023). Comparison of Distal Radius Fracture Outcomes in Older Adults Stratified by Chronologic vs. Physiologic Age Managed With Casting vs. Surgery. JAMA Netw. Open.

[28] Tulipan J.E., Lechtig A., Rozental T.D., Harper C.M. (2022). “Age Is Just a Number”: Distal Radius Fractures in Patients Over 75. Hand.

[29] Cornell C.N. (2003). Internal Fracture Fixation in Patients With Osteoporosis. J. Am. Acad. Orthop. Surg..

[30] Clayton R.A., Gaston M.S., Ralston S.H., Court-Brown C.M., McQueen M.M. (2009). Association between decreased bone mineral density and severity of distal radial fractures. J. Bone Jt. Surg. Am..

[31] Tuaño K.R., Fisher M.H., Lee N., Khatter N.J., Le E., Washington K.M., Iorio M.L. (2023). Analysis of Postoperative Distal Radius Fracture Outcomes in the Setting of Osteopenia and Osteoporosis for Patients with Comorbid Conditions. J. Hand Surg. Glob. Online.

[32] Ruffilli A., Manzetti M., Cerasoli T., Barile F., Viroli G., Traversari M., Salamanna F., Fini M., Faldini C. (2022). Osteopenia and sarcopenia as potential risk factors for surgical site infection after posterior lumbar fusion: A retrospective study. Microorganisms.

[33] FitzPatrick S.K., Casemyr N.E., Zurakowski D., Day C.S., Rozental T.D. (2012). The Effect of Osteoporosis on Outcomes of Operatively Treated Distal Radius Fractures. J. Hand Surg. Am..

[34] Arora R., Gabl M., Gschwentner M., Deml C., Krappinger D., Lutz M. (2009). A Comparative Study of Clinical and Radiologic Outcomes of Unstable Colles Type Distal Radius Fractures in Patients Older Than 70 Years: Nonoperative Treatment Versus Volar Locking Plating. J. Orthop. Trauma.

[35] Ostergaard P.J., Hall M.J., Rozental T.D. (2019). Considerations in the Treatment of Osteoporotic Distal Radius Fractures in Elderly Patients. Curr. Rev. Musculoskelet. Med..

[36] Ring D., Jupiter J.B. (2005). Treatment of osteoporotic distal radius fractures. Osteoporos. Int..

[37] Testa G., Vescio A., Di Masi P., Bruno G., Sessa G., Pavone V. (2019). Comparison between surgical and conservative treatment for distal radius fractures in patients over 65 years. J. Funct. Morphol. Kinesiol..

[38] Boymans T.A., van Helden S., Kessels A., Broeke R.T., Brink P.R.G. (2009). Bone mineral density is not correlated with one-year functional outcome in distal radial fractures: A preliminary study. Eur. J. Trauma Emerg. Surg..

[39] Cooper A.M., Wood T.R., Scholten Ii D.J., Carroll E.A. (2022). Nonsurgical management of distal radius fractures in the elderly: Approaches, risks and limitations. Orthop. Res. Rev..

[40] Ruzicka A., Kaiser P., Schmidle G., Benedikt S., Kastenberger T., Arora R. (2023). Die konservative Behandlung der distalen Radiusfraktur [Conservative treatment of distal radial fractures]. Oper. Orthop. Traumatol..

[41] Gorter E.A., Gerretsen B.M., Krijnen P., Appelman-Dijkstra N.M., Schipper I.B. (2020). Does osteoporosis affect the healing of subcapital humerus and distal radius fractures?. J. Orthop..

[42] Lee H.W., Kim K.T., Lee S., Yoon J.-H., Kim J.-Y. (2024). Fracture severity and triangular fibrocartilage complex injury in distal radius fractures with or without osteoporosis. J. Clin. Med..

[43] Paasikallio K., Sund R., Miettinen S., Kauranen S., Sorsa H., Kröger H., Sirola J. (2023). Intra- and inter-observer agreement in distal radius fracture dislocation measurement of casting position. Acta Orthop..

[44] Yousaf I.S., Guarino G.M., Sanghavi K.K., Rozental T.D., Means K.R., Giladi A.M. (2022). Development of osteopenia during distal radius fracture recovery. J. Hand Surg. Glob. Online.

[45] Shin Y.H., Hong W.K., Kim J., Gong H. (2020). Osteoporosis care after distal radius fracture reduces subsequent hip or spine fractures: A 4-year longitudinal study. Osteoporos. Int..

[46] Aynardi M., Ilyas A. (2013). Pharmacologic management of osteoporosis. J. Hand Surg. Am..

[47] Freedman K.B., Kaplan F.S., Bilker W.B., Strom B.L., Lowe R.A. (2000). Treatment of osteoporosis: Are physicians missing an opportunity?. J. Bone Jt. Surg. Am..

[48] Falk S.S.I., Richter M., Schröder J., Böhme S., Mittlmeier T. (2024). Pre-existing osteoporosis and serum vitamin D levels in patients with distal radius fractures: Are we missing something?. Arch. Orthop. Trauma Surg..

